# A giant teratoma in the anterior mediastinum found prenatally: A case report

**DOI:** 10.3389/fped.2023.1128947

**Published:** 2023-03-13

**Authors:** Yuntian Su, Ailixiati Alifu, Haifan Wang, Shibin Lin, Xiaoman Su, Yong Wei, Ying Wan, Renwei Chen

**Affiliations:** ^1^ Department of Cardiothoracic Surgery, Hainan Women and Children’s Medical Center, Haikou, China; ^2^ Department of Ultrasound, Hainan Women and Children’s Medical Center, Haikou, China; ^3^ Department of Radiology, Hainan Women and Children’s Medical Center, Haikou, China; ^4^ Department of Ultrasound, The First Affiliated Hospital of Hainan Medical College, Haikou, China

**Keywords:** mediastinal tumor, teratoma, prenatal diagnosis, case report, cardiothoracic surgery

## Abstract

Prenatal anterior mediastinal teratomas are rare. Anterior mediastinal teratomas can cause edema during the perinatal period. Color Doppler ultrasonography and chest computed tomography (CT) are of great value in diagnosing neonatal anterior mediastinal teratomas. Here, we report a case of prenatally diagnosed neonatal anterior mediastinal teratoma. After birth, transthoracic echocardiography and chest enhanced CT showed a large solid mass in the pericardial cavity. Owing to compression of the heart, the tumor was completely removed 1 day after birth, and cardiopulmonary bypass was performed. Pathology results indicated an immature teratoma (Grade I). At 9-month follow-up, the patient remained in good overall condition without observed recurrences.

## Introduction

Teratomas are a type of germ cell tumor, and the anterior mediastinum is a predilection site for teratomas. Teratomas account for approximately 1%–10% of anterior mediastinal germ cell tumors (1). The incidence of mediastinal teratoma is much lower in children than in adults, at a ratio of approximately 1:5 (2). In neonates and even in fetuses, anterior mediastinal teratomas are rare.

In clinical diagnosis, the signs and symptoms of neonates with anterior mediastinal teratomas are often not characteristic. Prenatal color Doppler ultrasound can detect fetal anterior mediastinal tumors; postnatal cardiac ultrasonography can determine the cystic and solid nature of the tumor; and enhanced computed tomography (CT) can clearly show the density, calcification, and other tumor characteristics, as these are essential to diagnose teratoma (3). In clinical treatment, complete tumor resection is the best solution, and the prognosis of this disease is satisfactory, with a high long-term survival rate.

## Case presentation

**Figure 1:**
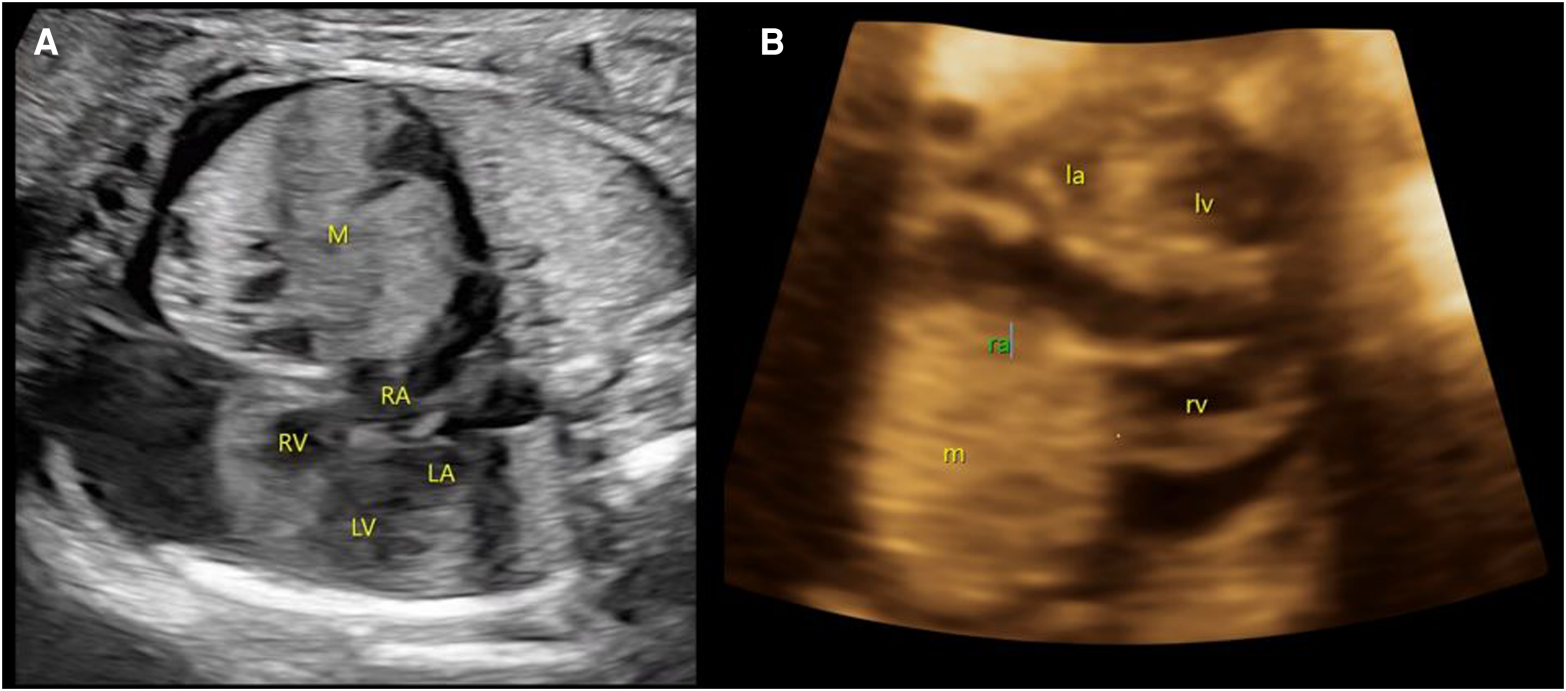
Prenatal ultrasound images. (**A**) At 26 weeks of gestation, a mixed mass (M) in the pericardium of the fetus with size approximately 27 × 26 mm with pericardial effusion is demonstrated. (**B**) At 37 weeks of gestation, a slightly strong echo mass (m) can be seen in the right pericardial cavity, with a range of approximately 37 × 36 mm. M/m, mass; RA/ra, right atrium; RV/rv, right ventricle; LA/La, left atrium; LV/Lv, left ventricle.

A male neonate was prenatally referred to our hospital for an anterior mediastinal mass. Four-dimensional ultrasound at 26 weeks of gestation revealed a mixed mass in the pericardium of the fetus with pericardial effusion (Figure 1). The cardiothoracic surgeon at our hospital suggested that there was no indication for prenatal intervention and recommended repeated ultrasound every other week, noting that surgery may be urgently required on the basis of echocardiogram after birth. At 37 gestational weeks, fetal echocardiography at our hospital showed a mass of approximately 37 × 36 mm in the right pericardial cavity.

The child was born at our hospital at 39 weeks of gestation and was admitted to the neonatal intensive care unit. He was born to a mother who was gravida 4, para 2 (G4P2), and he had a birth weight of 3,500 g, body length of 51 cm, head circumference of 34 cm, and chest circumference of 33 cm. The Apgar score was 9-9-9 (1 point deducted for skin color). On examination, he was afebrile, with a pulse rate of 132 beats/min, respiration rate of 42 breaths/min, and blood pressure of 75/42 mmHg. A tumor marker test showed that carbohydrate antigen 125 (CA125) was 57.5 µ/ml (normal range is less than 35 µ/ml), carbohydrate antigen 199 (CA199) was 200.6 µ/ml (normal range is less than 27 µ/ml), and alpha-fetoprotein (AFP) was more than 1,210 ng/ml (normal range in neonates is between 50 and 100,000 ng/ml).

**Figure 2:**
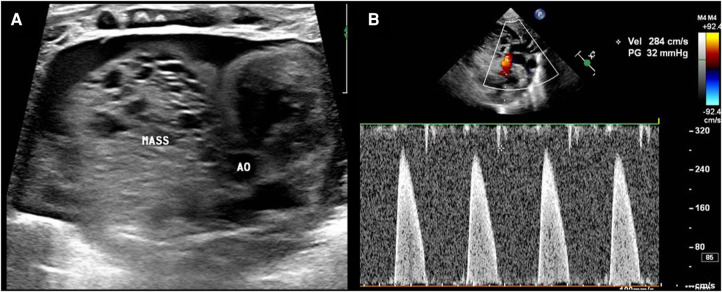
Postnatal ultrasound images. (**A**) The mass (MASS) is close to the ascending aorta (AO), and the boundary is not clear. (**B**) The mass compresses the ascending aorta, and the velocity in the ascending aorta reaches approximately 2.8 m/s.

**Figure 3:**
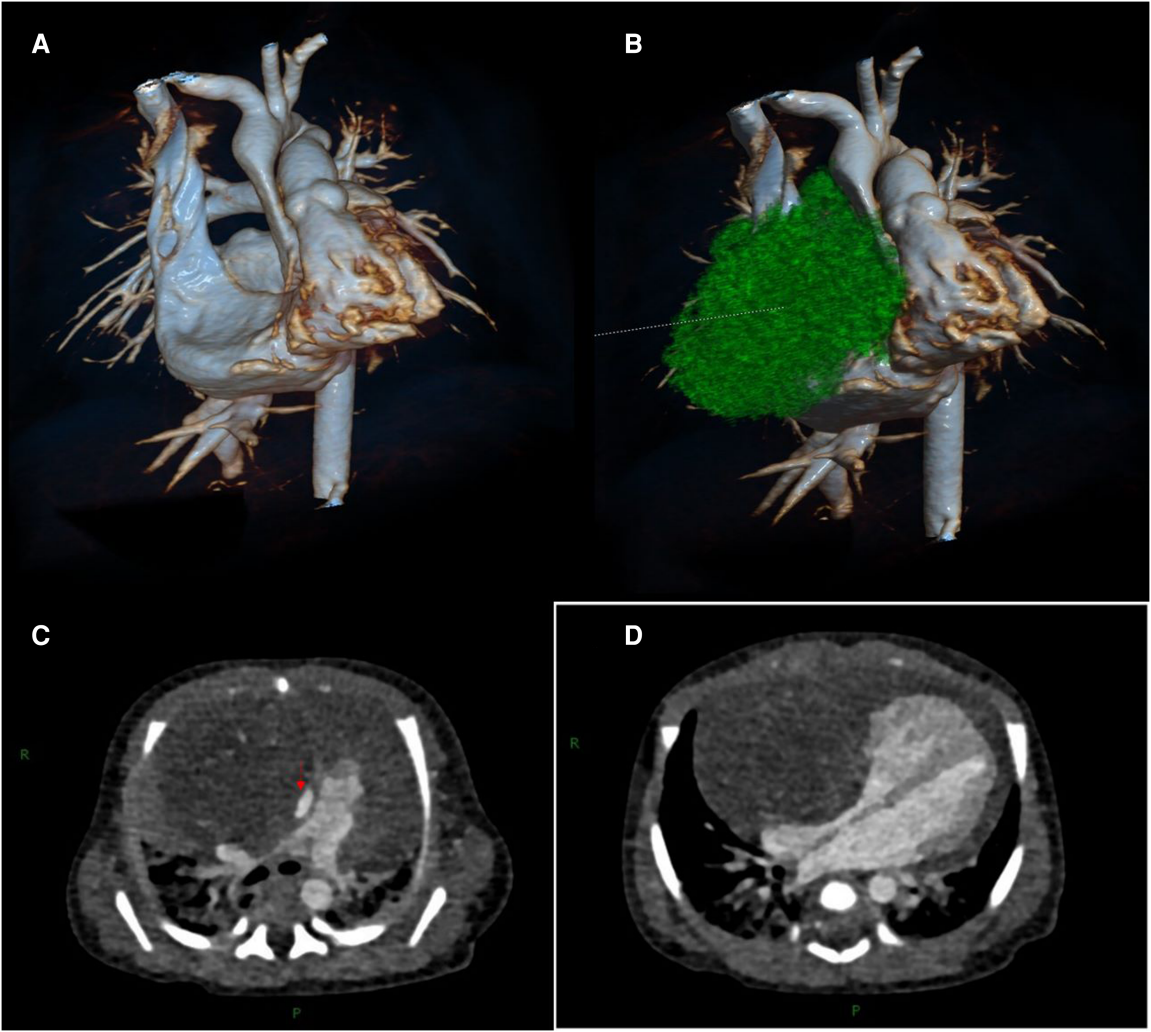
Cardiac computed tomography angiography images. (**A, B**) Three-dimensional modeling images show a huge mass in the right front of the heart, which severely compresses the ascending aorta and right atrium. (**C**) The aorta (red arrow) is compressed and flattened by the mass. (**D**) The mass compresses the right atrium.

Transthoracic echocardiography (TTE) showed a slightly hyperechoic mass in the right pericardial cavity, approximately 40 × 37 mm in size (Figure 2). The mass was close to the bottom of the heart and was compressing the ascending aorta, with a clear boundary and uneven echo. Patent ductus arteriosus and atrial septal left-to-right small shunts were also observed. For better evaluation and confirmation of the diagnosis, cardiac computed tomography angiography (CTA) was performed, and a slightly low-density mass was seen outside the right atrium, with a size of approximately 44.3 × 39.2 × 42.3 mm (Figure 3). High-density strip calcifications were observed at the edge of the mass. There was an unclear boundary and mild enhancement of the lesion. There were multiple small blood vessel shadows inside, with blood supply originating from the ascending aorta (approximately 1.0 mm in diameter); the ascending aorta was evidently compressed and flattened, the heart was displaced to the left, and a large amount of pericardial effusion was seen. Chest CT scan revealed neonatal pneumonia.

**Figure 4:**
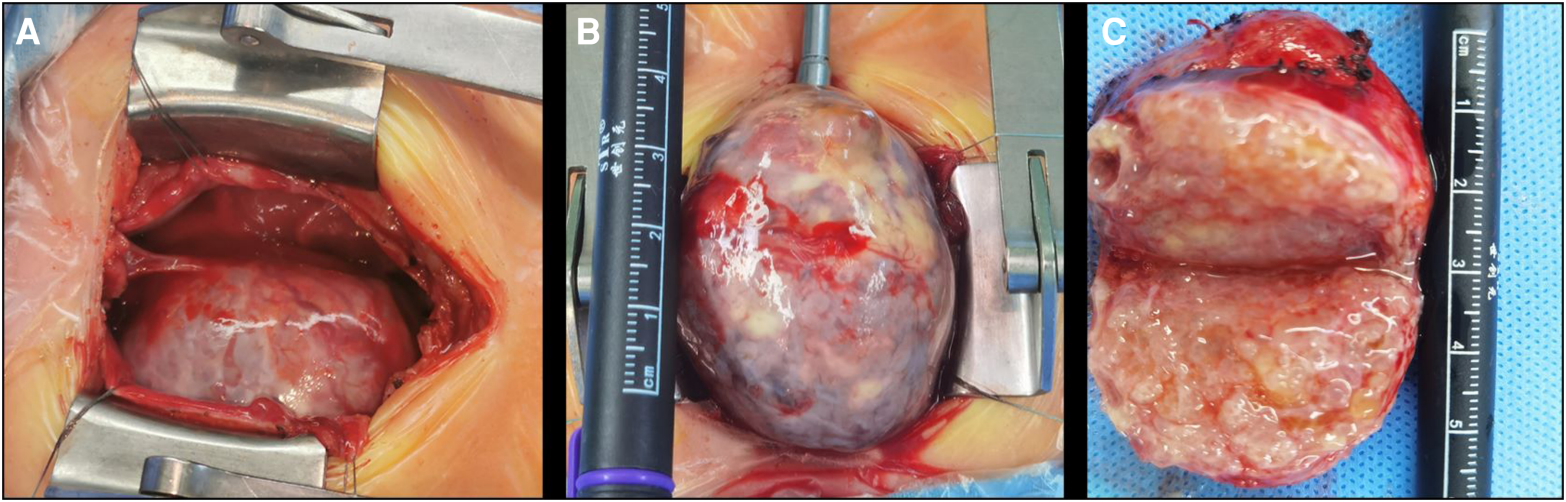
Intraoperative view. (**A**) The appearance of a mass after thoracotomy; (**B**) the appearance of a partially free mass placed outside the thoracic cavity; and (**C**) polycystic fat in the cross section after dissection of the mass.

Based on the above findings, the patient was diagnosed with an anterior mediastinal tumor. Considering the compression of the heart and aorta, surgery was indicated for the child. During the operation, a large solid mass, approximately 44 mm in size, was observed outside the right atrium, tightly connected to the right anterior wall of the ascending aorta. The mass had displaced the heart to the left, and the superior vena cava was compressed and posteriorly displaced (Figure 4). Blood pressure fluctuated frequently during dissociation and the mass was closely connected to the anterior wall of the aorta with unclear boundaries. Considering the risk of major bleeding or cardiac arrest, cardiopulmonary bypass was performed. The adhesion of the mass to the aorta was carefully loosened and the mass was completely removed. A small feeding artery was observed on the anterior wall of the aorta, which was sutured. The operation time was 3 h and 3 min; cardiopulmonary bypass time was 30 min, and intraoperative blood loss was 60 ml. Postoperative pathological examination was performed; this indicated that the mass was composed of a pseudolamellar ciliated columnar epithelium, a mucous columnar epithelium, a digestive gland, a few squamous epithelia, focal cartilage, smooth muscle tissue, and brain tissue. Therefore, the mass was diagnosed as an immature teratoma in the mediastinum (Grade I).

The child was postoperatively transferred to the cardiac intensive care unit. Arrhythmia occurred, and electrocardiogram showed polygenic premature ventricular contractions and paroxysmal ventricular tachycardia. After continuous infusion of lidocaine and 5 J synchronized electrical cardioversion, the patient was successfully cardioverted. The arrhythmia lasted for approximately 10 min, and might be attributable to the increased right ventricular return blood volume and right ventricular dilation after the operation.

Postoperative chest radiography and echocardiography revealed that the anterior mediastinal mass had been completely removed, the aorta was unobstructed, and cardiac function was good. Re-examination of the electrocardiogram showed no abnormalities. The neonate was extubated on the 5th postoperative day. Milk intake was increased to the normal range on the 12th postoperative day and the patient was transferred out of the intensive care unit. The patient’s weight increased from 3.5 to 3.65 kg and he was discharged on the 15th postoperative day.

The neonate was followed up regularly. TTE 9 months postoperatively showed normal cardiac function with good recovery.

## Discussion

Teratomas can be divided into congenital and acquired types; they occur mostly in the ovaries, testes, retroperitoneum, and mediastinum (4). Mediastinal teratomas in children most commonly occur congenitally, often without symptoms, and are often only accidentally discovered during chest radiography. Therefore, teratomas in infants and even neonates are relatively rarely found. In 2020, Senai et al. (3) reported on a case of a 3-month-old child with anterior mediastinal teratoma, considering this to be the youngest case reported in Uganda. Fetal mediastinal teratomas can be detected using prenatal ultrasound and can cause fetal hydrops. In 2019, Sihem and Houda (5) reported on a case of a fetus with mediastinal teratoma at 24 weeks of gestation that had induced severe fetal and placental hydrops. In the current case, the anterior mediastinal teratoma was found on antenatal four-dimensional ultrasonography during the fetal period, along with pericardial effusion, but the delivery was successful. However, prenatal ultrasound may misdiagnose mediastinal teratoma as congenital pulmonary airway malformation, diaphragmatic hernia, and other diseases.

Children with mediastinal teratomas may present with chest pain, dyspnea, or wheezing if the tumor is large and is severely compressing the surrounding tissues and organs. In the case of our patient, the right atrium and ascending aorta were compressed by the teratoma. Pericardial effusion and neonatal pneumonia were present, which might be caused by the teratoma.

AFP concentration is increased in children with germ cell tumors and is of great significance in the diagnosis (3), treatment, and follow-up of malignant teratomas. However, in the neonatal period, there is a physiological increase in AFP, with levels being generally higher than 2,000 ng/ml. Therefore, the value of neonatal AFP for the diagnosis and treatment of teratoma is limited. In this case, the patient’s AFP level was >1,210 ng/ml.

In terms of imaging, ultrasonography is of great value in the diagnosis of mediastinal teratomas because of the absence of ionizing radiation. Ultrasound can be used to identify cystic and solid masses and to check for pleural and pericardial effusion. In this case, ultrasonography showed that the mass was solid, with pericardial effusion. Chest CT has high sensitivity and practicability. A plain CT scan can show the material composition of the mass, such as calcification. CTA can show the blood supply of the mass and its relationship with the surrounding important blood vessels, thus helping in the formulation of surgical plans. For the differential diagnosis, mediastinal teratoma should be distinguished from congenital pulmonary airway malformation (CPAM) and diaphragmatic hernia. In this case, the CT findings showed mild enhancement of the lesions, and the blood supply vessel originated from the ascending aorta. There were no gas-containing vesicles or intestinal structures. Therefore, a diagnosis of CPAM or diaphragmatic hernia was not considered. Imaging examinations showed that the mass was severely compressing the right atrium, superior vena cava, and ascending aorta, and a large amount of effusion was observed in the pericardium. This supported the clinical diagnosis and provided clear indications for surgery.

Complete surgical resection of mediastinal teratomas is the optimal treatment option. The purpose of the surgery is as follows: first, to clarify the nature of the mass; second, to relieve the compression of the mass on the surrounding tissue, thereby avoiding possible serious complications. However, anterior mediastinal tumors may exert different degrees of compression on important tissues, such as the heart, large blood vessels, and trachea, which may lead to the risk of cardiac arrest during general anesthesia in children. Therefore, it has been reported that extracorporeal membrane oxygenation or cardiopulmonary bypass technology may be used to provide perioperative life support for children (6). In our patient, the mass was closely connected to the right anterior wall of the ascending aorta. The mass had displaced the heart to the left, and the superior vena cava had moved posteriorly under pressure. To avoid the risk of massive hemorrhage and cardiac arrest caused by aortic rupture, cardiopulmonary bypass was established intraoperatively. The surrounding adhesions were carefully dissociated, the normal tissue structure was preserved as far as possible, and the teratoma was completely removed. The teratoma in this case was solid, and the incised section showed that it was mainly a fat-like material, but no clear hair, bone, or other substances were found. Regarding the differential diagnosis, the degree of differentiation of immature teratomas is low, and the structure is not clear. Therefore, pathological examination is warranted to determine the diagnosis. In this case, the mass consisted of epithelial, focal cartilage, smooth muscle, and brain tissues. Consequently, the mass was diagnosed as an immature teratoma.

After the operation, the patient recovered and was discharged from the hospital without any related symptoms such as dyspnea or edema.

Patients with benign or low-grade teratomas have a good prognosis after complete removal of the teratoma (7). The patient in this case is still under long-term follow-up.

## Conclusion

Anterior mediastinal teratomas found prenatally and treated during the neonatal period are very rare. They are usually discovered incidentally during postnatal physical examination. Perinatal ultrasonography and CT play an important role in the diagnosis of anterior mediastinal teratomas. Based on reports in the literature and our experience, complete surgical resection is the optimal solution for the treatment of anterior mediastinal teratomas. If the mediastinal teratoma is closely related to the great vessels of the heart, extracorporeal surgery may be considered to avoid serious risks such as massive bleeding and cardiac arrest. Integrated diagnosis and provision of pre- and postnatal treatment is of great importance for early detection, prompt treatment, and good prognosis in cases of anterior mediastinal teratoma.

## Data Availability

The original contributions presented in the study are included in the article/Supplementary Material; further inquiries can be directed to the corresponding author.
